# Preoperative Metabolic Syndrome and HDL-C Level Predict the Prognosis of Patients Following Radical Cystectomy: A Propensity Score Matching Study

**DOI:** 10.3389/fonc.2022.833305

**Published:** 2022-04-05

**Authors:** Zenan Liu, Hai Bi, Wei He, Xuehua Zhu, Jide He, Min Lu, Jian Lu

**Affiliations:** ^1^ Department of Urology, Peking University Third Hospital, Beijing, China; ^2^ Department of Pathology, Peking University Third Hospital, Beijing, China; ^3^ NHC Key Laboratory of Metabolic Cardiovascular Diseases Research, Ningxia Medical University, Yinchuan, China

**Keywords:** bladder cancer, radical cystectomy, metabolic syndrome, high-density lipoprotein cholesterol (HDL-C), survival outcome, propensity score matching

## Abstract

**Objective:**

To investigate the prognostic significance of metabolic syndrome (MetS) and its components in patients with bladder cancer (BCa) treated with radical cystectomy (RC).

**Methods:**

A total of 335 BCa patients who underwent RC between 2004 and 2019 at Peking University Third Hospital (PUTH) were analyzed retrospectively. The Kaplan-Meier method with the log-rank test was performed to assess overall survival (OS) and progression-free survival (PFS). Univariate and multivariate Cox proportional hazard models were conducted to identify the prognostic factors of OS and PFS before and after propensity score matching (PSM).

**Results:**

Enrolled patients were allocated into two groups according to the presence or absence of MetS (n=84 MetS vs n=251 non-MetS), and 82 new matched pairs were identified to balance the baseline characteristics after 1:1 PSM. In the Kaplan-Meier analysis, MetS was associated with better OS (P=0.031) than the group without MetS. In addition, a body mass index (BMI) ≥ 25 was associated with better OS (P=0.011) and PFS (P=0.031), while low high-density lipoprotein cholesterol (HDL-C) was associated with worse OS (P=0.033) and PFS (P=0.010). In all patients, multivariate Cox analysis showed that hemoglobin, pathologic tumor stage and lymph node status were identified as independent prognostic factors for both OS and PFS, while age, MetS and HDL-C were independent prognostic factors only for OS. Reproducible results of multivariate analysis can still be observed in propensity matched patients. The results of further subgroup analysis revealed that the association of MetS with increased OS (P=0.043) and BMI ≥25 with increased OS (P=0.015) and PFS (P=0.029) was observed in non-muscle invasive bladder cancer (NMIBC) patients.

**Conclusions:**

MetS was independently associated with better OS in BCa patients after RC, and HDL-C was the only component of MetS that was independently associated with worse OS. MetS and HDL-C may become reliable prognostic biomarkers of OS in BCa patients after RC to provide individualized prognostication and assist in the formulation of clinical treatment strategies.

## Introduction

Bladder cancer (BCa) is one of the most common malignancies of the genitourinary system and is a significant cause of morbidity and mortality worldwide. It was estimated that BCa accounted for 83,730 new cases of cancer and 17,200 cancer-related deaths in 2021 (1). Urothelial carcinoma (UC) is the most common histologic type, approximately 75% of patients present with non-muscle invasive bladder cancer (NMIBC)while 25% with muscle invasive bladder cancer (MIBC), and 10-20% of cases of NMIBC will progress to MIBC at diagnosis (2). Radical cystectomy (RC) remains the standard treatment for non-metastatic MIBC (3) and high risk NMIBC (4). Despite significant advancement in surgical techniques and increasing application of multimodal treatment approaches, the long-term survival outcome of BCa patients after RC is not satisfactory, and the 5-year disease-specific survival after RC is consistently 50-60% (5, 6). To improve the survival outcome, the assessment of reliable prognostic factors could be conducive to guiding clinical decision-making and patient consultation, such as tumor stage, lymph node status, lymphovascular invasion (LVI), pathologic grade (7), lymphocyte-to-monocyte (LMR) (8) and Vesical Imaging-Reporting and Data System (VI-RADS) score (9). Among them, tumor stage and lymph node status remain the dominant pathologic predictors for recurrence and survival. However, BCa with similar stage and grade may present significantly different clinical outcomes after RC unexpectedly (10). Therefore, it is necessary to identify additional appropriate prognostic factors to help in preoperative risk stratification and survival prediction.

Recently, there is increasing interest in describing the extent of the impact of metabolic changes on cancer development and progression, particularly with regard to metabolic syndrome (MetS) (11–14). MetS is a complex disorder characterized by a series of metabolic disturbances including abdominal obesity, hyperglycemia, high blood pressure, hypertriglyceridemia, and low high-density lipoprotein cholesterol (HDL-C) (15), all of which are independently associated with an increased risk of cancer (16–18), and their influence on survival outcome has been confirmed in a variety of cancers as well, such as liver cancer (19), gastric cancer (20), breast cancer (21) and colon cancer (11). In addition, MetS is closely associated with a variety of genitourinary diseases as well (22, 23). As for BCa, current studies focus more on the potential association between MetS and an increased risk of BCa (24). In contrast, data on the association between MetS and survival outcomes in BCa, such as overall survival, cancer-specific survival and disease recurrence, are extremely limited and unproven (25). Similarly, only a few studies have evaluated the relationship between each MetS component and the survival outcomes of BCa in detail (26, 27).

Thus, in view of the significant role of MetS in tumor prognosis, this study was designed to explore the prognostic significance of MetS and its components in BCa patients treated with RC.

## Materials and Methods

### Study Population

**Figure 1:**
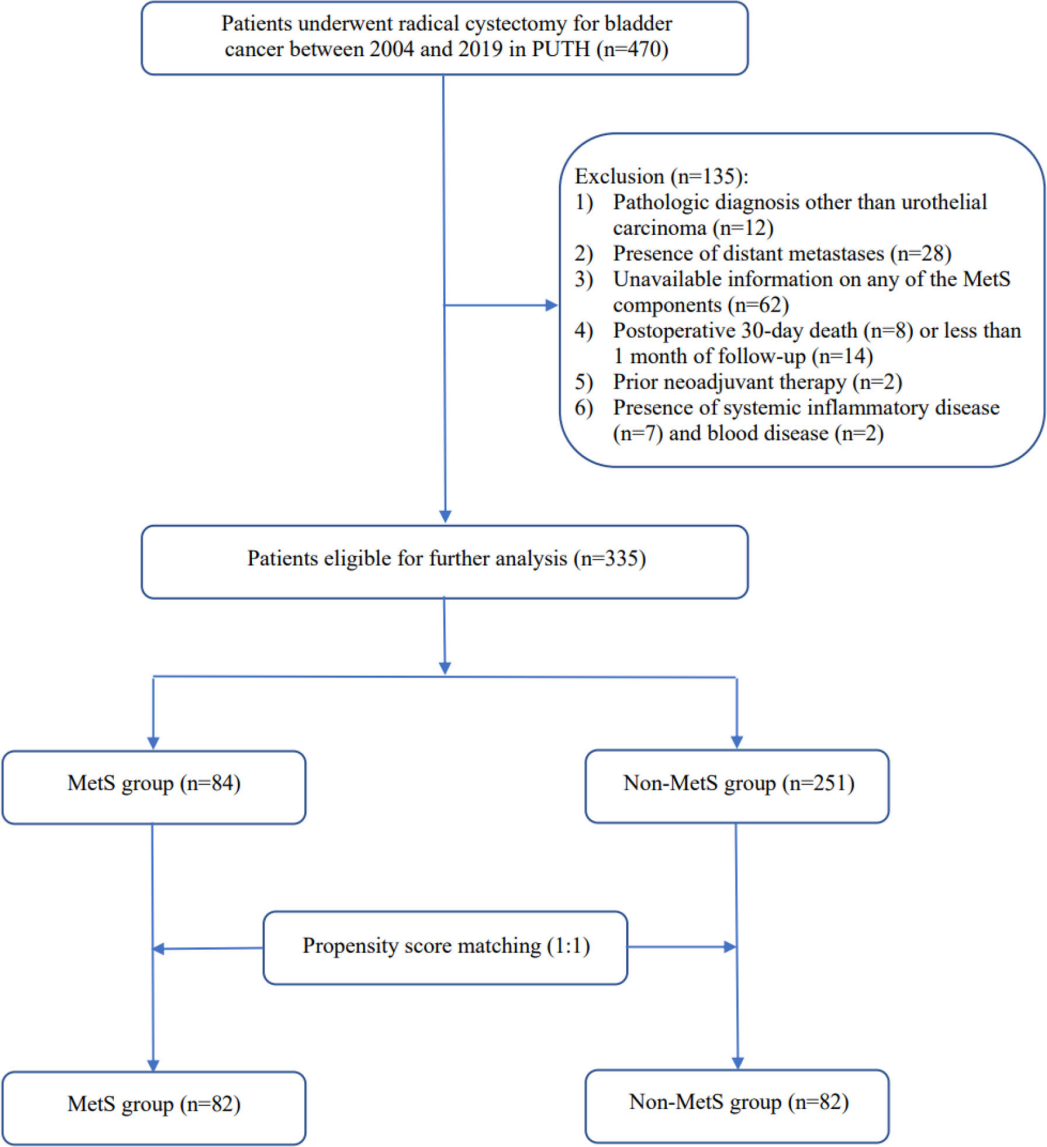
Flow chart of patient selection.

After obtaining the approval of the Institutional Review Board for the Protection of Human Subjects, we used the BCa database from the Department of Urology at Peking University Third Hospital (PUTH)for our analysis. A total of 470 consecutive BCa patients treated with RC between 2004 and 2019 at PUTH were included in the study. Comprehensive clinicopathological information was reviewed and collected for each patient. Patients were excluded from the study based on the following criteria: pathologic diagnosis other than urothelial carcinoma (n=12), distant metastatic disease at the time of RC (n=28), unavailable information on any of the MetS components (n=62), postoperative 30-day death (n=8) or less than 1 month of follow-up (n=14), prior neoadjuvant therapy (n=2), and presence of systemic inflammatory disease (n=7) and blood disease (n=2). This resulted in 335 BCa patients eligible for further analysis, the process of patient selection is shown in **Figure 1**.

### Data Collection

The clinical and pathological variables of the enrolled patients were retrospectively collected from the database, including: age, gender, body mass index (BMI), hypertension, hyperglycemia, hypertriglyceridemia, high-density lipoprotein cholesterol (HDL-C), current smoking, hemoglobin (Hg), pathologic tumor stage (pT), lymph node status (pN), pathologic grade, concomitant carcinoma *in situ* (CIS), variation and adjuvant therapy. All surgical specimens after RC were processed according to standard pathological procedures. Genitourinary pathologists assigned tumor pathologic grade and clinical stage according to the 2004 WHO/International Society of Urologic Pathologists classification of bladder urothelial cancer and the 2017 TNM staging system of the AJCC, respectively.

### Metabolic Syndrome Criteria

Patients were classified as MetS according to the diagnostic criteria from Chinese Medical Association Diabetes Society in 2004 (28) with at least three of the following four components: (i) overweight and/or obesity: body mass index (BMI) ≥ 25kg/m^2^; (ii) hyperglycemia: fasting plasma glucose ≥6.1mmol/L (110 mg/dL) and/or 2-hr postprandial plasma glucose ≥7.8mmol/L (140 mg/dL), or drug treatment for diagnosed diabetes mellitus; (iii) hypertension: blood pressure ≥140/90 mmHg or drug treatment for diagnosed hypertension; (iv) dyslipidemia: fasting serum triglyceride (TG) level ≥1.7mmol/L (150 mg/dL) and/or fasting serum high-density lipoprotein cholesterol (HDL-C) <0.9mmol/L (35 mg/dL) in male and <1.0mmol/L (39 mg/dL) in female.

### Follow-Up

In general, the patient underwent postoperative clinical and radiological follow-up following conventional institutional protocols, included quarterly sessions for the first two years, semiannually sessions for the next two years, and then annual follow-up thereafter. The primary study outcomes included overall survival (OS) and progression-free survival (PFS). OS was defined as the time from the day of surgery to the last follow-up or death due to any cause. PFS was defined as the time from the beginning of treatment to the observation of disease progression or death due to any cause.

### Statistical Analysis

According to the data distribution, continuous variables are presented as medians and interquartile ranges (IQRs), and categorical variables are expressed as counts and frequencies. Comparisons of the differences between MetS patients and non-MetS patients were performed using Student’s t test for continuous variables and the χ^2^ test or Fisher’s test for categorical variables. We reduced the influence of data deviation and confounding variables between the patients in the MetS and non-MetS groups by using the method of propensity score matching (PSM) to obtain matched data. Matching was conducted at a 1:1 fixed ratio with a caliper value of 0.05 by using the variables of age, gender, current smoking, hemoglobin, pT stage, pN status, pathologic grade, CIS, variation and adjuvant therapy. OS and PFS were estimated using standard Kaplan-Meier methods. The log-rank test was applied for the statistical comparison between survival curves. Univariate and multivariate analyses were performed using the Cox proportional hazards model to assess the correlation between MetS and individual components and survival outcomes, and the results were presented as hazards ratios (HRs) and 95% confidence intervals (95% CIs). All significant variables with a P value < 0.10 in the univariate analysis were incorporated into the subsequent multivariate analysis to identify the independent prognostic factors. All statistical analyses were performed using IBM SPSS Statistics 26.0. Two-sided P values <0.05 were considered statistically significant.

## Results

### Patient Characteristics

A total of 335 patients treated with RC were included in the study and they were divided into two groups based on the presence or absence of MetS (n=84 MetS vs n=251 non-MetS). The overall prevalence of each of the various MetS components was 38.2% for obesity, 39.1% for hypertension, 34.9% for hyperglycemia, 30.4% for hypertriglyceridemia and 27.5% for low HDL-C. To balance the baseline and reduce the impact of potential confounding factors, PSM was performed at a 1:1 fixed ratio, and finally we obtained 82 new matched pairs. After matching, the clinicopathologic characteristics between patients in the MetS and non-MetS groups were well-balanced except for individual components of MetS (BMI, P <0.001; hypertension, P <0.001; hyperglycemia, P <0.001; hypertriglyceridemia, P <0.001; low HDL-C, P <0.001). The clinicopathologic characteristics of the all patients and propensity matched patients are shown in **Table 1**.

**Table 1:** Clinicopathological characteristics of the all patients and propensity matched patients.

Characteristics	All patients (n=335)			Propensity matched patients (n=164)		
	MetS (n=84)	Non-MetS (n=251)	P value	MetS (n=82)	Non-MetS (n=82)	P value
**Age (years), median (IQR)**	68 (59-73)	68 (60-75)	0.895	68.5 (59-73.3)	69 (61.8-76.3)	0.497
**Gender, n (%)**			0.044			0.319
**Male**	64 (76.2%)	215 (85.7%)		64 (78.0%)	69 (84.1%)	
**Female**	20 (23.8%)	36 (14.3%)		18 (22.0%)	13 (15.9%)	
**BMI (kg/m2), n (%)**			<0.001			<0.001
**<25**	16 (19.0%)	191 (76.1%)		16 (19.5%)	61 (74.4%)	
**≥25**	68 (81.0%)	60 (23.9%)		66 (80.5%)	21 (25.6%)	
**Hypertension, n (%)**			<0.001			<0.001
**No**	20 (23.8%)	184 (73.3%)		20 (24.4%)	60 (73.2%)	
**Yes**	64 (76.2%)	67 (26.7%)		62 (75.6%)	22 (26.8%)	
**Hyperglycemia, n (%)**			<0.001			<0.001
**No**	20 (23.8%)	198 (78.9%)		19 (23.2%)	69 (84.1%)	
**Yes**	64 (76.2%)	53 (21.1%)		63 (76.8%)	13 (15.9%)	
**Hypertriglyceridemia, n (%)**			<0.001			<0.001
**No**	31 (36.9%)	202 (80.5%)		30 (36.6%)	64 (78.0%)	
**Yes**	53 (63.1%)	49 (19.5%)		52 (63.4%)	18 (22.0%)	
**Low HDL-C, n (%)**			<0.001			<0.001
**No**	42 (50.0%)	201 (80.1%)		41 (50%)	64 (78.0%)	
**Yes**	42 (50.0%)	50 (19.9%)		41 (50%)	18 (22.0%)	
**Current Smoking, n (%)**			0.514			0.724
**No**	63 (75.0%)	179 (71.3%)		61 (74.4%)	59 (72.0%)	
**Yes**	21 (25.0%)	72 (28.7%)		21 (25.6%)	23 (28.0%)	
**Hg (g/L), median (IQR)**	137 (124-148)	132 (117-145)	0.150	137 (123-149)	134 (118-149)	0.588
**pT Stage, n (%)**			0.663			0.510
**≤T2**	52 (61.9%)	162 (64.5%)		52 (63.4%)	56 (68.3%)	
**T3-4**	32 (38.1%)	89 (35.5%)		30 (36.6%)	26 (31.7%)	
**pN Status, n (%)**			0.452			0.277
**Negative**	68 (81.0%)	212 (84.5%)		67 (81.7%)	72 (87.8%)	
**Positive**	16 (19.0%)	39 (15.5%)		15 (18.3%)	10 (12.2%)	
**Pathologic Grade, n (%)**			0.559			0.755
**LG**	5 (6.0%)	11 (4.4%)		5 (6.1%)	6 (7.3%)	
**HG**	79 (94.0%)	240 (95.6%)		77 (93.9%)	76 (92.7%)	
**Variation, n (%)**			0.993			0.717
**Absent**	78 (92.9%)	233 (92.8%)		77 (93.9%)	79 (96.3%)	
**Present**	6 (7.1%)	18 (7.2%)		5 (6.1%)	3 (3.7%)	
**Concomitant CIS, n (%)**			0.425			0.711
**Absent**	64 (76.2%)	180 (71.7%)		62 (75.6%)	64 (78.0%)	
**Present**	20 (23.8%)	71 (28.3%)		20 (24.4%)	18 (22.0%)	
Adjuvant Therapy*, n (%)			0.328			0.292
**No**	74 (88.1%)	210 (83.7%)		72 (87.8%)	76 (92.7%)	
**Yes**	10 (11.9%)	41 (16.3%)		10 (12.2%)	6 (7.3%)	

BCa, bladder cancer; BMI, body mass index; CIS, carcinoma in situ; HDL-C, high density lipoprotein cholesterol; Hg, hemoglobin; HG, high grade; IQR, interquartile range; LG, low grade; MetS, metabolic syndrome; pN, pathologic node stage; pT, pathologic tumor stage; RC, radical cystectomy.

^*^Adjuvant radiotherapy and/or adjuvant chemotherapy.

### Survival Outcomes of OS and PFS

In total, the median follow-up period was 34.0 months (interquartile range: 13.0-64.0 months), with a total of 27 (32.1%) patients who died and 34 (40.5%) who developed disease progression in the MetS group, and 106 (42.2%) who died and 118 (47.0%) who developed disease progression in the non-MetS group. The median OS time was 46.4 months, and the 5-year OS probabilities for the MetS group and non-MetS group were 70.2% and 60.2%, respectively. The median PFS time weas 36.9 months, and the 5-year PFS probabilities for the MetS group and non-MetS group were 63.1% and 55.8%, respectively.

### Univariate and Multivariate Analyses for OS and PFS in All Patients

Univariate analyses revealed that age, BMI, hemoglobin, pathologic tumor stage and lymph node status were associated with OS; age, HDL-C, hemoglobin, pathologic tumor stage, lymph node status and adjuvant therapy were associated with PFS (**Table 2**). After adjusting for potential confounders by multivariate Cox regression analysis, age (P=0.011), MetS (P=0.005), HDL-C (P=0.006), hemoglobin (P<0.001), pathologic tumor stage (P<0.001) and lymph node status (P=0.001) were identified as independent prognostic factors for OS; hemoglobin (P<0.001), pathologic tumor stage (P=0.001) and lymph node status (P<0.001) were identified as independent prognostic factors for PFS (**Table 2**).

**Table 2:** Univariate and multivariate analysis of prognostic factors using the Cox proportional hazards model for OS and PFS in all patients.

Variables	OS				PFS			
	Univariate analysis		Multivariate analysis		Univariate analysis		Multivariate analysis	
	HR (95% CI)	P value	HR (95% CI)	P value	HR (95% CI)	P value	HR (95% CI)	P value
Age (years)	1.033 (1.014-1.052)	0.001	1.026 (1.006-1.046)	0.011	1.022 (1.006-1.040)	0.009	1.009 (0.991-1.027)	0.317
Gender								
Male	Ref				Ref			
Female	0.975 (0.612-1.556)	0.917			0.962 (0.622-1.489)	0.862		
Metabolic Syndrome								
No	Ref		Ref		Ref			
Yes	0.677 (0.441-1.041)	0.076	0.466 (0.272-0.798)	0.005	0.776 (0.527-1.143)	0.199		
BMI								
<25	Ref		Ref		Ref		Ref	
≥25	0.676 (0.470-0.974)	0.035	1.273 (0.809-2.004)	0.297	0.731 (0.523-1.023)	0.068	0.854 (0.601-1.214)	0.380
Hypertension								
No	Ref				Ref			
Yes	0.837 (0.587-1.194)	0.327			0.897 (0.645-1.247)	0.516		
Hyperglycemia								
No	Ref				Ref			
Yes	1.035 (0.723-1.481)	0.852			1.005 (0.718-1.406)	0.978		
Hypertriglyceridemia								
No	Ref				Ref			
Yes	0.854 (0.578-1.263)	0.430			0.953 (0.667-1.360)	0.789		
Low HDL-C								
No	Ref		Ref		Ref		Ref	
Yes	1.412 (0.981-2.033)	0.064	1.719 (1.165-2.537)	0.006	1.504 (1.073-2.107)	0.018	1.410 (0.999-1.990)	0.051
Current Smoking								
No	Ref				Ref			
Yes	0.980 (0.670-1.433)	0.916			0.916 (0.639-1.312)	0.633		
Hg (g/L)	0.976 (0.968-0.983)	<0.001	0.980 (0.971-0.988)	<0.001	0.977 (0.970-0.984)	<0.001	0.980 (0.972-0.988)	<0.001
pT Stage								
≤T2	Ref		Ref		Ref		Ref	
T3-4	2.927 (2.066-4.147)	<0.001	2.055 (1.406-3.005)	<0.001	2.935 (2.119-4.065)	<0.001	1.896 (1.322-2.718)	0.001
pN Status								
Negative	Ref		Ref		Ref		Ref	
Positive	2.689 (1.786-4.049)	<0.001	2.147 (1.381-3.336)	0.001	3.402 (2.345-4.937)	<0.001	2.685 (1.765-4.084)	<0.001
Pathologic Grade								
LG	Ref		Ref		Ref		Ref	
HG	2.346 (0.861-6.387)	0.095	1.057 (0.372-3.004)	0.917	2.182 (0.888-5.360)	0.089	1.182 (0.464-3.009)	0.726
Variation								
Absent	Ref				Ref			
Present	0.715 (0.315-1.624)	0.422			0.599 (0.264-1.356)	0.219		
Concomitant CIS								
Absent	Ref				Ref			
Present	0.770 (0.501-1.184)	0.233			0.816 (0.554-1.202)	0.304		
Adjuvant Therapy*								
No	Ref				Ref		Ref	
Yes	0.814 (0.495-1.341)	0.419			1.520 (1.011-2.286)	0.044	0.929 (0.599-1.441)	0.742

BCa, bladder cancer; BMI, body mass index; CI, confidence interval; CIS, carcinoma in situ; HDL-C, high density lipoprotein cholesterol; Hg, hemoglobin; HG, high grade; HR, hazard ratio; IQR, interquartile ranges; LG, low grade; OS, overall survival; PFS, progression-free survival; pN, pathologic node stage; pT, pathologic tumor stage; Ref, reference.

^*^Adjuvant radiotherapy and/or adjuvant chemotherapy.

### The Effect of MetS and Its Components on OS and PFS in Propensity Matched Patients

**Figure 2:**
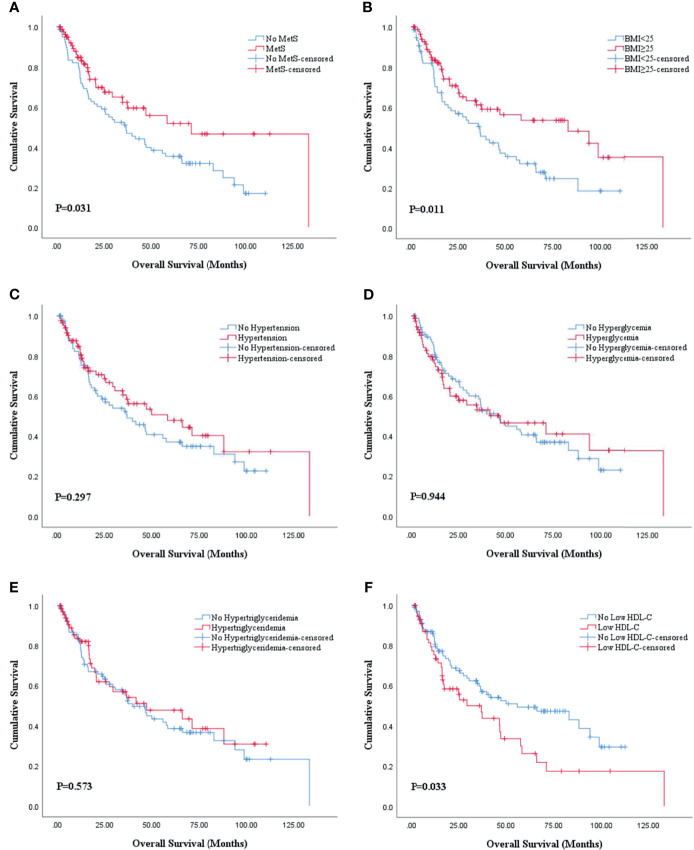
Kaplan-Meier survival analysis of OS stratified by MetS and its components after PSM. **(A)** MetS and non-MetS; **(B)** BMI <25 and BMI ≥25; **(C)** hypertension and no hypertension; **(D)** hyperglycemia and no hyperglycemia; **(E)** hypertriglyceridemia and no hypertriglyceridemia; **(F)** low HDL-C and no low HDL-C. BMI, body mass index; HDL-C, high density lipoprotein cholesterol; MetS, metabolic syndrome; OS, overall survival; PSM, propensity score match.

**Figure 3:**
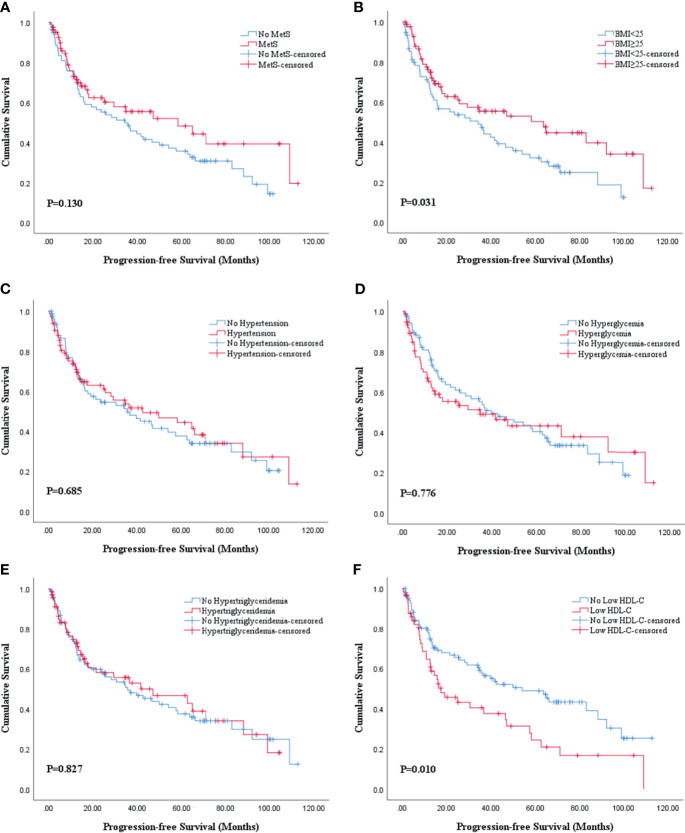
Kaplan-Meier survival analysis of PFS stratified by MetS and its components after PSM. **(A)** MetS and non-MetS; **(B)** BMI <25 and BMI ≥25; **(C)** hypertension and no hypertension; **(D)** hyperglycemia and no hyperglycemia; **(E)** hypertriglyceridemia and no hypertriglyceridemia; **(F)** low HDL-C and no low HDL-C. BMI, body mass index; HDL-C, high density lipoprotein cholesterol; MetS, metabolic syndrome; PFS, progression-free survival; PSM, propensity score match.

The Kaplan-Meier analysis and log-rank test revealed that there was statistical significance in both OS and PFS curves for BMI and HDL-C, and statistical significance in only OS curves for MetS. BMI ≥25 was associated with better OS (P=0.011; **Figure 2B**) and PFS (P=0.031; **Figure 3B**) while low HDL-C was associated with worse OS (P=0.033; **Figure 2F**) and PFS (P=0.010; **Figure 3F**). In addition, MetS was also associated with better OS (P=0.031) compared with non-MetS (**Figure 2A**). There was no statistical significance for other individual components of MetS in the OS (**Figures 2C–E**) and PFS (**Figures 3A, C–E**) curves.

Univariate analyses revealed that age, BMI, HDL-C, hemoglobin, pathologic tumor stage and lymph node status were all associated with OS and PFS while MetS was only associated with OS (**Table 3**). In order to ensure the assessment values of prognostic factors was consistent with that before PSM, we also performed multivariate Cox regression analysis after PSM. As a result, age (P=0.019), MetS (P=0.001), HDL-C (P=0.011), hemoglobin (P<0.001), pathologic tumor stage (P=0.001) and lymph node status (P=0.017) were identified as independent prognostic factors for OS. Hemoglobin (P=0.004), pathologic tumor stage (P=0.002) and lymph node status (P=0.007) were identified as independent prognostic factors for PFS (**Table 3**).

**Table 3:** Univariate and multivariate analysis of prognostic factors using the Cox proportional hazards model for OS and PFS in propensity matched patients.

Variables	OS				PFS			
	Univariate analysis		Multivariate analysis		Univariate analysis		Multivariate analysis	
	HR (95% CI)	P value	HR (95% CI)	P value	HR (95% CI)	P value	HR (95% CI)	P value
Age (years)	1.046 (1.020-1.073)	0.001	1.035 (1.006-1.065)	0.019	1.038 (1.013-1.063)	0.002	1.019 (0.993-1.046)	0.148
Gender								
Male	Ref				Ref			
Female	0.904 (0.499-1.636)	0.738			0.958 (0.550-1.670)	0.881		
Metabolic Syndrome								
No	Ref		Ref		Ref			
Yes	0.600 (0.375-0.959)	0.033	0.361 (0.195-0.669)	0.001	0.717 (0.465-1.105)	0.132		
BMI								
<25	Ref		Ref		Ref		Ref	
≥25	0.566 (0.362-0.885)	0.013	1.225 (0.684-2.195)	0.494	0.631 (0.414-0.961)	0.032	0.698 (0.451-1.081)	0.107
Hypertension								
No	Ref				Ref			
Yes	0.791 (0.508-1.231)	0.299			0.917 (0.604-1.393)	0.685		
Hyperglycemia								
No	Ref				Ref			
Yes	1.016 (0.649-1.590)	0.944			1.063 (0.696-1.624)	0.776		
Hypertriglyceridemia								
No	Ref				Ref			
Yes	0.877 (0.554-1.387)	0.574			0.953 (0.619-1.467)	0.827		
Low HDL-C								
No	Ref		Ref		Ref		Ref	
Yes	1.617 (1.035-2.527)	0.035	1.861 (1.150-3.013)	0.011	1.733 (1.137-2.642)	0.011	1.522 (0.985-2.350)	0.058
Current Smoking								
No	Ref				Ref			
Yes	0.983 (0.606-1.594)	0.943	Ref		0.877 (0.549-1.403)	0.585		
Hg (g/L)	0.974 (0.964-0.984)	<0.001	0.978 (0.966-0.990)	<0.001	0.976 (0.967-0.986)	<0.001	0.984 (0.973-0.995)	0.004
pT Stage								
≤T2	Ref		Ref		Ref		Ref	
T3-4	2.938 (1.885-4.579)	<0.001	2.312 (1.432-3.734)	0.001	3.037 (1.990-4.636)	<0.001	2.079 (1.319-3.278)	0.002
pN Status								
Negative	Ref		Ref		Ref		Ref	
Positive	3.263 (1.874-5.683)	<0.001	2.043 (1.138-3.668)	0.017	3.502 (2.089-5.872)	<0.001	2.147 (1.227-3.756)	0.007
Pathologic Grade								
LG	Ref		Ref		Ref			
HG	2.684 (0.842-8.560)	0.095	1.160 (0.343-3.921)	0.811	2.251 (0.819-6.184)	0.116		
Variation								
Absent	Ref				Ref			
Present	0.238 (0.033-1.710)	0.154			0.450 (0.111-1.831)	0.265		
Concomitant CIS								
Absent	Ref				Ref			
Present	0.985 (0.569-1.704)	0.957			1.005 (0.604-1.672)	0.985		
Adjuvant Therapy*								
No	Ref				Ref			
Yes	0.877 (0.422-1.825)	0.726			1.443 (0.765-2.724)	0.257		

BCa, bladder cancer; BMI, body mass index; CI, confidence interval; CIS, carcinoma in situ; HDL-C, high density lipoprotein cholesterol; Hg, hemoglobin; HG, high grade; HR, hazard ratio; IQR, interquartile ranges; LG, low grade; OS, overall survival; PFS, progression-free survival; pN, pathologic node stage; PSM, propensity score match; pT, pathologic tumor stage; Ref, reference.

^*^Adjuvant radiotherapy and/or adjuvant chemotherapy.

Further subgroup analyses were performed stratified by T stage (NMIBC vs MIBC). The results revealed that the association of MetS with increased OS (P=0.043) and BMI ≥25 with increased OS (P=0.015) and PFS (P=0.029) were observed in NMIBC patients. In contrast, there were no significant differences in MetS and its individual components in the OS and PFS curves of MIBC patients (**Supplementary Figures 1–4**).

## Discussion

In the present single-center study, we investigated the impact of MetS and its components on the prognosis of BCa patients who underwent RC. We balanced out differences in clinicopathological characteristics between MetS and non-MetS patients and explored the influence of other potential risk factors by using PSM and multivariate Cox regression analysis. Our study found that MetS was independently associated with better OS in BCa patients after RC, and HDL-C was the only component of MetS that was independently associated with worse OS. We further performed detailed subgroup analyses stratified by tumor stage, and the results revealed that the presence of MetS and BMI ≥ 25 were protective factors for the survival of NMIBC patients. To the best of our knowledge, this is the first study to explore whether MetS or its components influence survival outcomes in BCa patients treated with RC, which might provide preliminary evidence and direction for future research in this area.

Our results highlight the necessary for more investigation into the potential molecular mechanisms underlying our findings. A variety of mechanisms have been proposed to explain the role of MetS in cancers including regulation of the insulin-like growth factor-1 (IGF-1) pathway, existence of hyperinsulinemia and insulin resistance, process of adipokine production, angiogenesis promotion, glucose malutilization, and oxidative stress/DNA damage, which can synergistically increase the cancer risk rather than just individual components (29, 30). Insulin can bind and activate the IGF-1 receptor and promote mitosis by triggering downstream pathways to act as a growth factor (31). Increased levels of insulin and IGF-1 can would promote tumors growth and progression by binding to the overexpressed insulin receptor in many cancers (32). At the same time, insulin resistance in patients with MetS can contribute to hyperinsulinemia, which enhances the activity of IGF by inhibiting the synthesis of IGF binding proteins (33). In addition to endocrine disorders, immuno-inflammatory responses such as adipose tissue releases proinflammatory cytokines such as TNF-α or IL-6, which promote angiogenesis and cell proliferation leading to rapid tumor growth (34). Besides, hyperglycemia is associated with mitochondrial malfunction, which leads to insufficient DNA repair and increases the production of reactive oxygen species (ROS) to raise oxidative stress damage (35). Therefore, there are complex prognostic effects in cancer patients due to the complex mechanisms between MetS and cancer.

The impact of MetS on cancer patient prognosis, including BCa, remains controversial. Several studies have illustrated that MetS is negatively associated with the survival outcomes of cancers. For instance, Hu D et al. discovered that the median survival time for MetS patients was significantly shorter than for non-MetS patients in a prospective study of 3012 gastric cancer patients (20). The result of Xu H et al. study also showed that MetS was an independent factor for decreased cancer-specific survival (CSS) in upper tract urothelial carcinoma (UTUC) patients (36). In contrast, Yang Y et al. (37) and Silva A et al. (11) both concluded that MetS was not a prognostic factor for OS or recurrence-free survival (RFS) in patients with colon cancer. Garg T et al. also found that there was no association between MetS and time to recurrence in a large, multi-institutional cohort of older patients with NMIBC (38). Interestingly, our results revealed that MetS was a favorable prognostic factor that was associated with better OS in patients with BCa after RC. Similar results have been seen in other cancer studies. Wen YS et al. also discovered that MetS was associated with improved survival in patients with resectable esophageal squamous cell carcinoma independently and significantly (39). Furthermore, Liu Z et al. found MetS to be an independent favorable prognostic factor of CSS in patients with localized renal cell carcinoma (RCC) (33). These results could be explained by the fact that patients with MetS were generally accompanied by a better nutrition status, which could reduce the risk of mortality caused by malnutrition. Good nutritional status could improve survival by enhancing immunity and providing high tolerance for long-term treatment (40). In addition, there are studies suggesting that the better survival outcomes of RC patients with MetS in our study might be the result of a beneficial role played by obesity, which is a vital constituent of MetS. Patients with higher BMI might have better nutritional status and a potential survival advantage (41).

Obesity is a major component of MetS and was considered to be associated with worse outcomes in BCa patients treated with RC. Dabi Y et al. showed that obesity increased the risk of recurrence and cancer-specific mortality in patients with NMIBC and MIBC (26). Chromecki TF et al. found that BMI ≥30 kg/m^2^ was an independent predictor of cancer recurrence, cancer-specific mortality and OS in patients treated with RC for UC of the bladder (42). However, inconsistent with previous observational studies, Kaplan-Meier analysis and log-rank test in the present study revealed that overweight and obese patients (BMI ≥ 25) showed a significantly more favorable survival outcome (OS: p = 0.011; PFS: p = 0.031) compared with normal weight patients (BMI < 25). Similar results were observed in NMIBC patients in further subgroup analysis. Other studies have reached similar conclusions to support our findings. Kwon T et al. reported that overweight patients who underwent RC had a better prognosis with decreased recurrence and cancer-specific mortality compared with normal BMI values in 714 Korean patients with both NMIBC and MIBC (43). At the same time, the result of univariate analysis in Xu X et al. study also noted that a significantly favorable decreased all-cause mortality in the higher BMI group (≥ 31.2 kg/m2) compared with a low BMI group (< 31.2 kg/m^2^) (44). These results suggest that, contrary to the popular viewpoint, obesity might confer a survival benefit in BCa patients treated with RC. However, the specific mechanism of obesity related to a protective function in BCa remains insufficiently clear. The potential protective mechanisms resulting from overweight may be due to the elevated levels of proinflammatory molecules (45) such as adiponectin, cytokines and leptin, which are produced by adipose tissue. Leptin plays an anti-tumor role by promoting the proliferation and activation of natural killer cells (46). In addition, in MIBC patients, lymphocytes exert effect of tumor suppression by combining with adipocytes to contribute to immune regulation, antigen recognition, and elimination of malignant cells. Periprostatic mature adipocytes could also release TGFβ1 upregulated connective tissue growth factor (CTGF) expression in prostate cancer cells favoring migration (47). Therefore, BMI may be a potential reliable predictor of prognosis in RC patients, but further well-controlled clinical research with large sample sizes regarding this topic are still warranted.

Similar to obesity, diabetes mellitus (DM) is also a strong single risk factor for MetS components, and was potentially positively associated with adverse survival outcomes in patients with BCa (48). One retrospective study based on 1,502 patients who underwent RC for MIBC and high-risk NMIBC showed that compared with nondiabetic patients, there a significantly increased risk of disease recurrence, cancer-specific mortality, and any-cause mortality in diabetic patients without metformin therapy (49). Ferro M et al. concluded that type 2 diabetes mellitus (T2DM) was a predictor of an increased risk of recurrence and progression in patients with primary T1HG/G3 NMIBC in a large multi-institutional cohort (50). Hwang EC et al. also reported that DM seems to be an independent predictor of RFS and PFS in NMIBC patients (51). In contrast, our results did not show a significant relationship between DM and the prognosis of RC patients. This inconsistency might be explained by the effect of DM medication (metformin or insulin) on the ultimate survival outcome. Metformin has been discovered to play effective antineolastic effect by inhibiting the mammalian target of AMP-activated protein kinase (AMPK)-dependent and liver kinase B1 (LKB1)-dependent rapamycin (mTOR) pathway (52). Several studies found that the use of metformin seems to be associated with better RFS or CSS in patients with BCa (27, 53). Furthermore, the results from Rieken M et al. also showed that DM patients who used metformin had similar oncological outcomes after RC compared with non-DM patients (49). Therefore, future researches are supposed to consider the drug treatment of DM and further explore the impact of DM on the prognosis of RC patients.

Unlike obesity and DM, the role of hypertension in development and progression of BCa has not been well investigated. No unified conclusion has been reached in existing studies concerning hypertension and the prognosis of BCa. In the study of Stocks T et al. (54), elevated blood pressure increased the incidence and mortality rate of BCa in men, whereas there is no significant association between hypertension and BCa has been found in other studies (48). The results of Anceschi U et al. showed that hypertension was not significantly associated with OS in patients treated with robot assisted radical cystectomy (55), which was consistent with our findings. Abnormal proliferation in vascular smooth muscle cells might be the important link between hypertension and cancer. However, more evidence is needed to clarify the correlation between hypertension and BCa, as well as the underlying mechanism.

In the process of analyzing the impact of dyslipidemia on the outcomes of BCa patients treated with RC, we found that preoperative low HDL- C was independent predictors of worse OS while hypertriglyceridemia was not associated with both in OS and PFS in RC patients, suggesting that low HDL-C might be the primary component contributing to the associations between MetS and adverse prognosis of RC patients. Existing evidence suggests that HDL-C represent cancer cell renewal and epiphenomenon of cancer-related inflammation, which is closely associated with cancer-related mortality and incidence. Low HDL-C might play a vital role in cancer progression by promoting proinflammatory cytokine production, inhibiting antioxidation and inducing apoptosis (56). In addition, the associations between low HDL-C and cancer prognosis in other studies have also drawn meaningful result echoing the above mechanism. The result of Li X et al. study illustrated that breast cancer patients with lower HDL-C levels [≤ 1.02 mmol/L (40 mg/dl)] had worse OS and disease-free survival (DFS) compared with those with higher HDL-C levels (57). Xu H et al. also found the potential connection between low HDL-C and worse OS, CSS and RFS in patients with UTUC (36). Therefore, HDL-C may be a favorable marker for prognostic prediction for RC patients, and further studies are still required to elucidate the role and investigate whether HDL-C targeted therapy would improve the survival outcomes of BCa patients after RC.

There are several limitations of the study that need to be acknowledged. First of all, this study is a single-center retrospective study, which has the inherent shortcoming of limited sample size and inevitable selection bias. Second, this study mainly focused on the Chinese population, which might result in ethnicity bias and affect the generalization of our results. The role of MetS and its components in BCa patients after RC in other races or ethnicities still remains to be explored. Third, we adopted BMI ≥ 25 kg/m^2^ to define obesity instead of the commonly used BMI ≥ 30 kg/m^2^or waist circumferences considering the particularity of Chinese population, which might lead to misclassification and affected the final results. Last but not least, we did not obtain relevant drug treatment concerning MetS components such as stains or metformin due to the lack of information. This may be an important source of bias, as these drugs might have an impact on survival outcomes.

## Conclusion

In conclusion, we found that MetS was independently associated with better OS in patients with BCa treated with RC, and HDL-C was the only component of MetS that was independently associated with worse OS. MetS and HDL-C may become reliable prognostic biomarkers of OS in BCa patients after RC to provide individualized prognostication and assist in the formulation of clinical treatment strategies. However, given the inherent limitations of this study, these results need to be further confirmed by adequately designed prospective studies with larger populations to provide a better conclusion.

## Data Availability Statement

The raw data supporting the conclusions of this article will be made available by the authors, without undue reservation.

## Ethics Statement

The studies involving human participants were reviewed and approved by the Peking University Third Hospital Medical Science Research Ethics Committee (No. M2018183). The patients/participants provided their written informed consent to participate in this study.

## Author Contributions

JL designed the research and controlled the structure and quality of the manuscript. ZL collected and analyzed the data and wrote this manuscript. HB, WH, XZ, and JH collected the data and helped in designing the study. ML was responsible for pathology data. All authors contributed to the article and approved the submitted version.

## Funding

This work was supported by grants from Beijing Natural Science Foundation (Z200027) and the National Natural Science Foundation of China (No.61871004).

## Conflict of Interest

The authors declare that the research was conducted in the absence of any commercial or financial relationships that could be construed as a potential conflict of interest.

## Publisher’s Note

All claims expressed in this article are solely those of the authors and do not necessarily represent those of their affiliated organizations, or those of the publisher, the editors and the reviewers. Any product that may be evaluated in this article, or claim that may be made by its manufacturer, is not guaranteed or endorsed by the publisher.
