# Supplementary material for: Preoperative Metabolic Syndrome and HDL-C Level Predict the Prognosis of Patients Following Radical Cystectomy: A Propensity Score Matching Study
Source: Front Oncol. 2022 Apr 5;12:833305. doi: 10.3389/fonc.2022.833305 (PMC9022107; doi:10.3389/fonc.2022.833305)
Supplement: Supplementary file 1 [file DataSheet_1.docx]

Supplementary Material

# Supplementary Figures

C

B

| **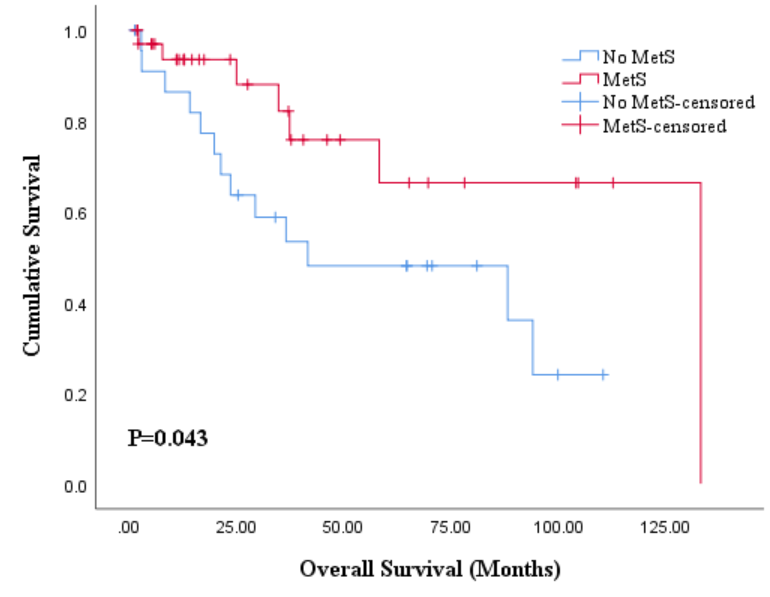**  A | **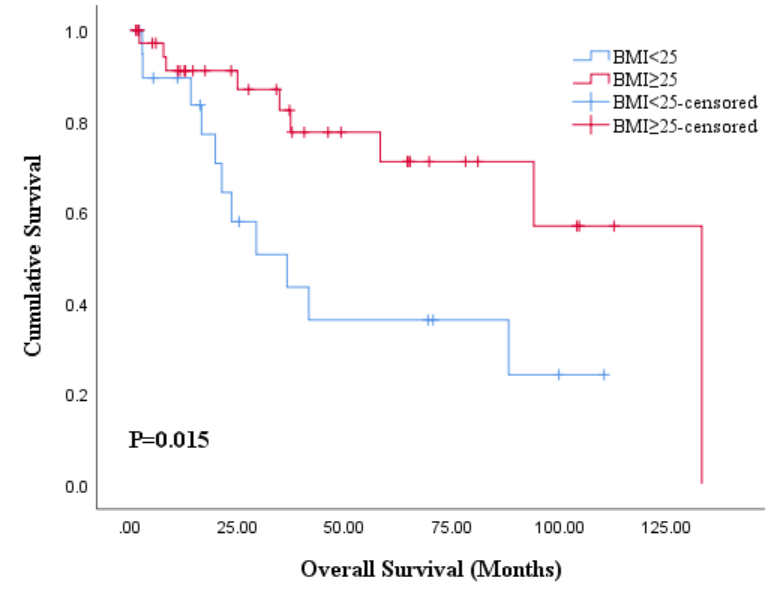**  D |
| --- | --- |
| **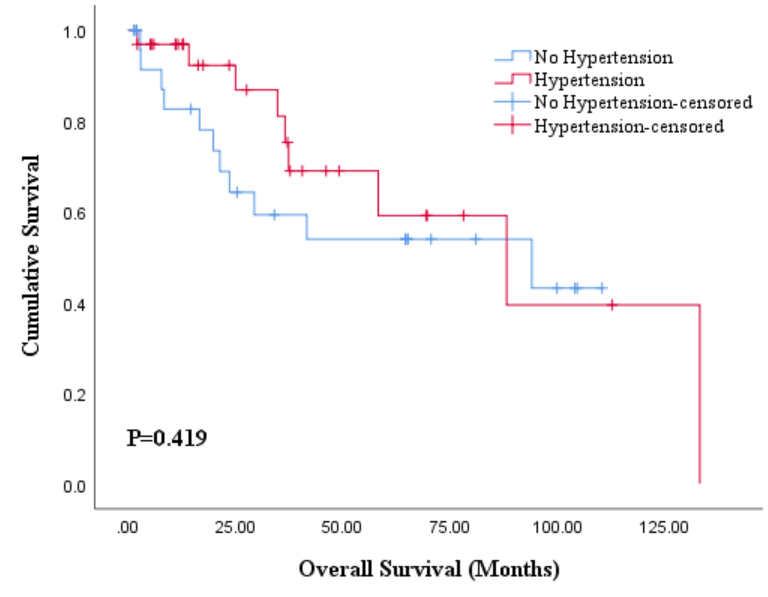** | **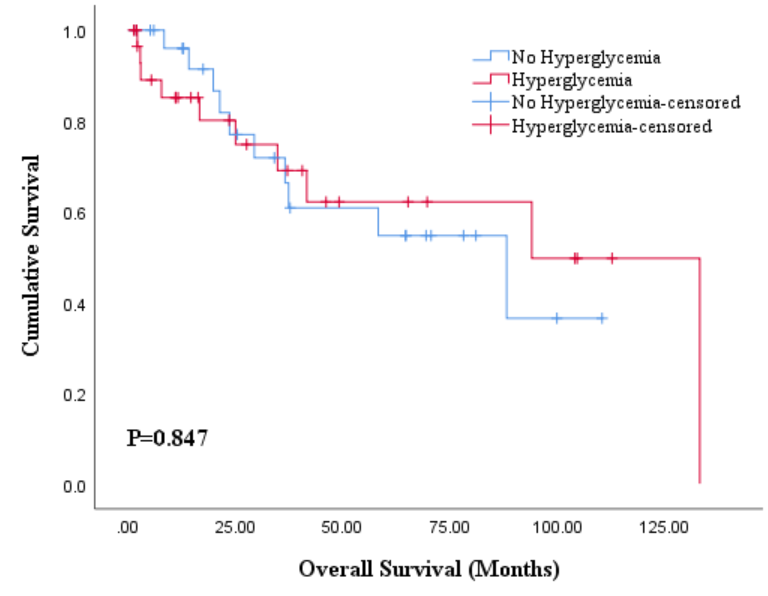** |
| **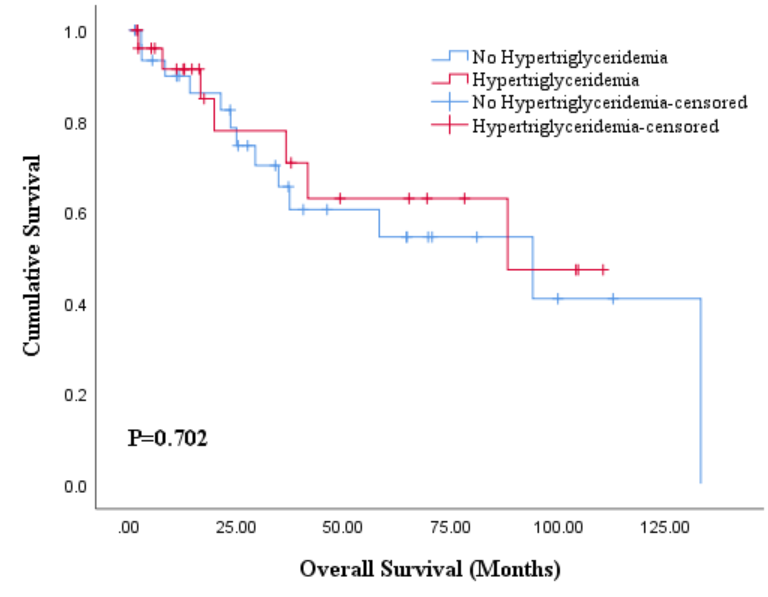** | **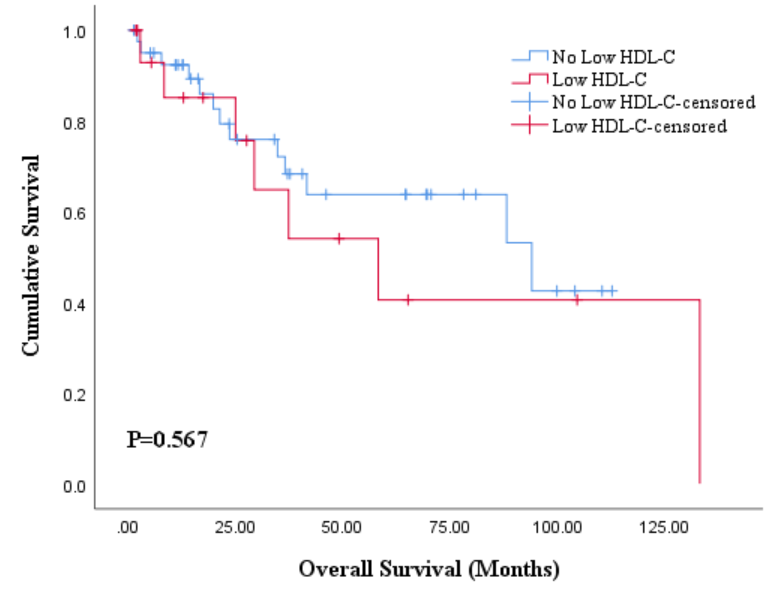** |

**Supplementary Figure 1** Kaplan-Meier survival analysis of the OS stratified by MetS and its components in NMIBC patients after PSM. **(A)** MetS and non-MetS; **(B)** BMI <25 and BMI ≥25; **(C)** hypertension and no hypertension; **(D)** hyperglycemia and no hyperglycemia; **(E)** hypertriglyceridemia and no hypertriglyceridemia; **(F)** low HDL-C and no low HDL-C. BMI, body mass index; HDL-C, high density lipoprotein cholesterol; MetS, metabolic syndrome; NMIBC, non-muscle invasive bladder cancer; OS, overall survival; PSM, propensity score match.

F

E

| **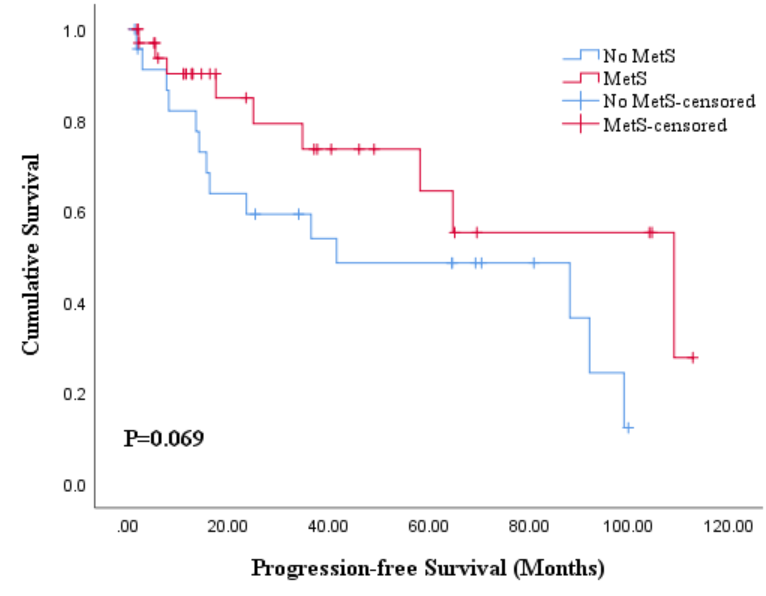**  A | **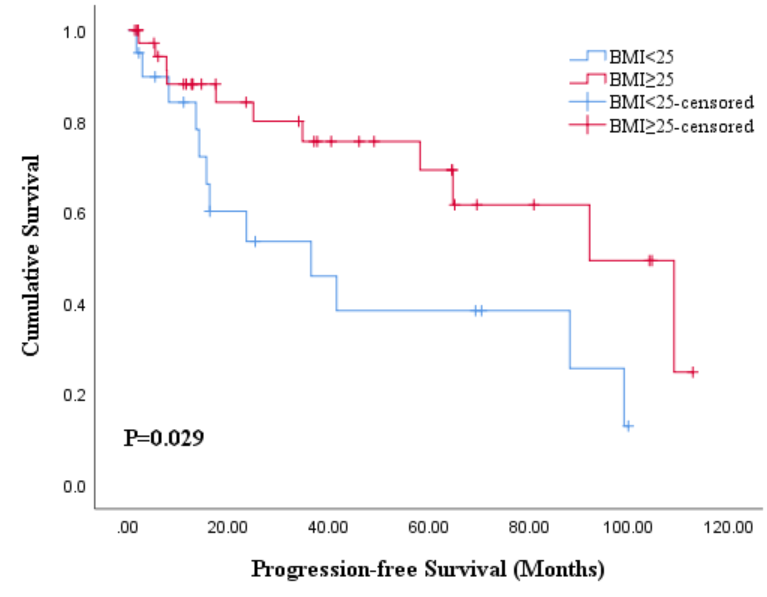**  B |
| --- | --- |
| **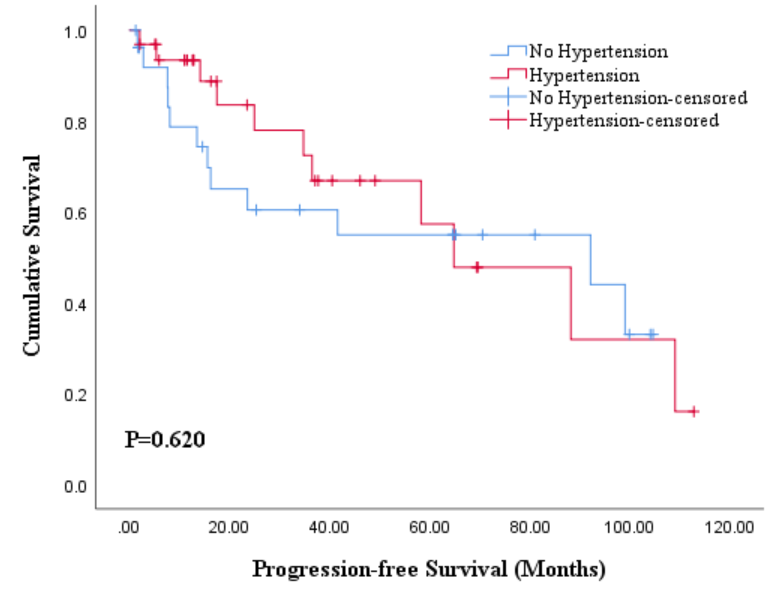**  D | **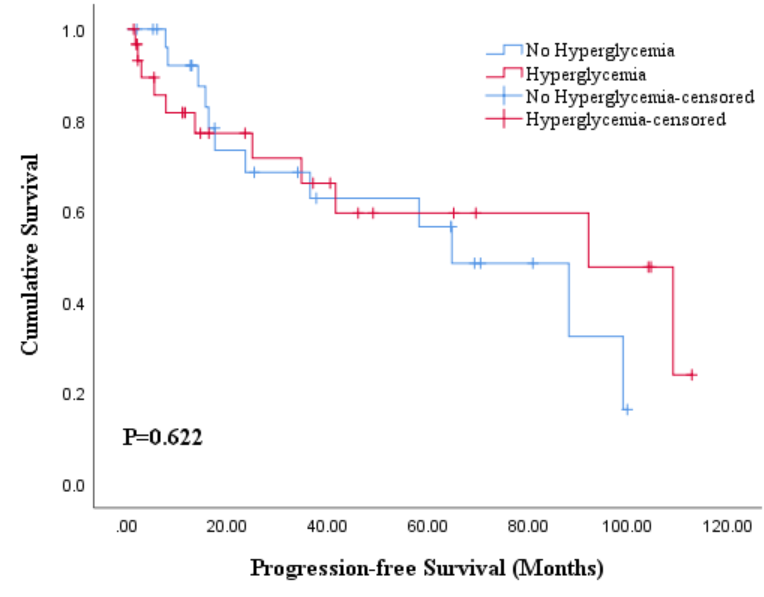**  F |
| **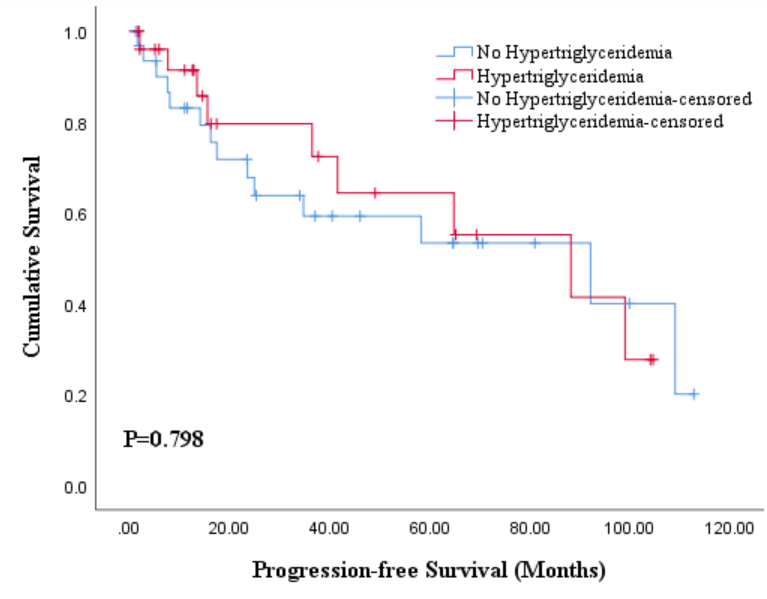**  E | **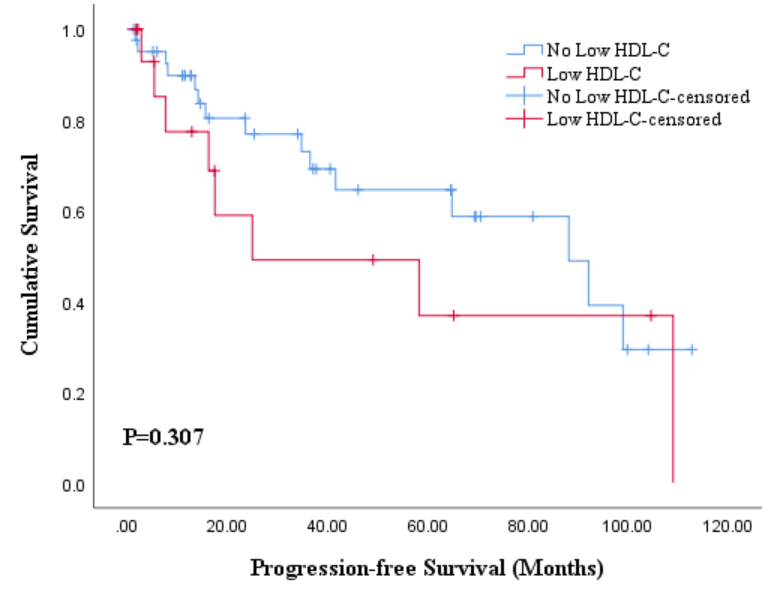** |

**Supplementary Figure 2** Kaplan-Meier survival analysis of the PFS stratified by MetS and its components in NMIBC patients after PSM. **(A)** MetS and non-MetS; **(B)** BMI <25 and BMI ≥25; **(C)** hypertension and no hypertension; **(D)** hyperglycemia and no hyperglycemia; **(E)** hypertriglyceridemia and no hypertriglyceridemia; **(F)** low HDL-C and no low HDL-C. BMI, body mass index; HDL-C, high density lipoprotein cholesterol; MetS, metabolic syndrome; NMIBC, non-muscle invasive bladder cancer; PFS, progression-free survival; PSM, propensity score match.

C

| **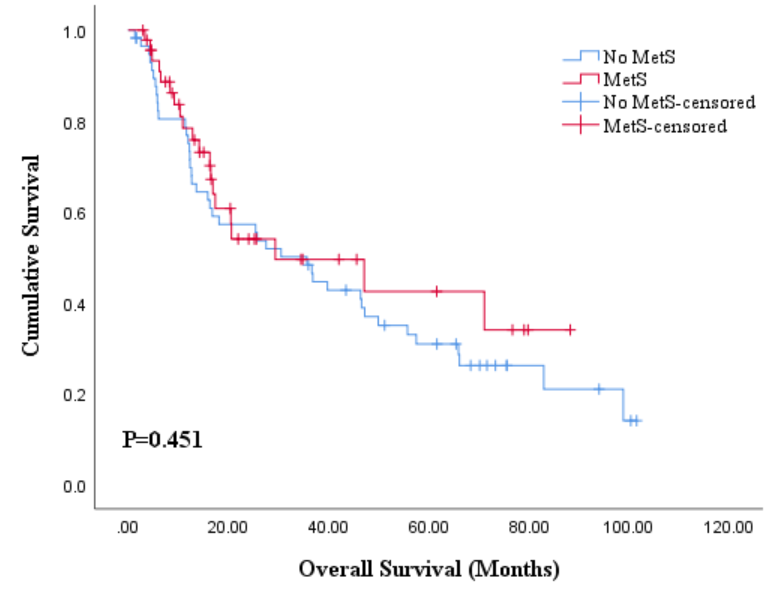**  C  B  A | **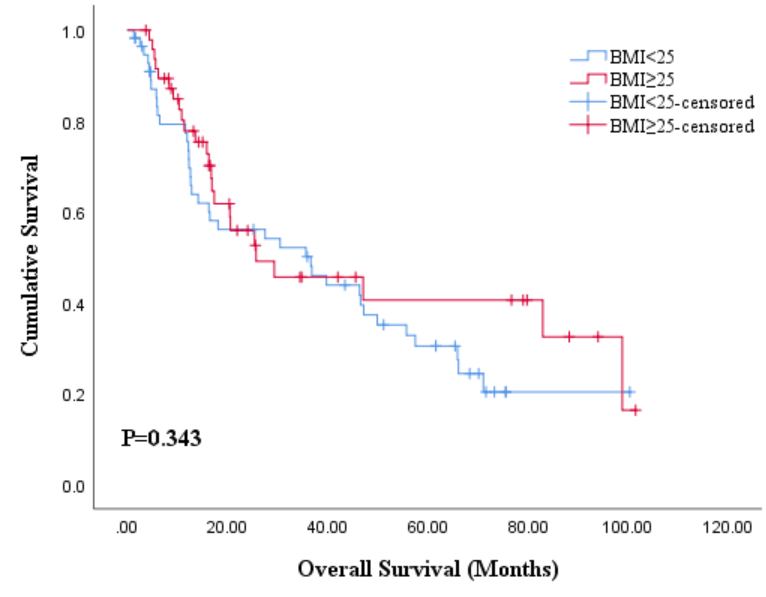** |
| --- | --- |
| **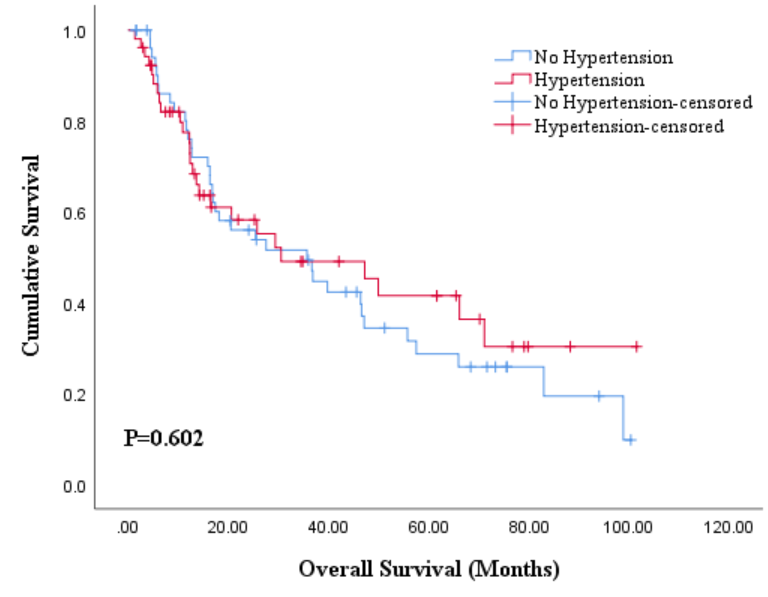** | **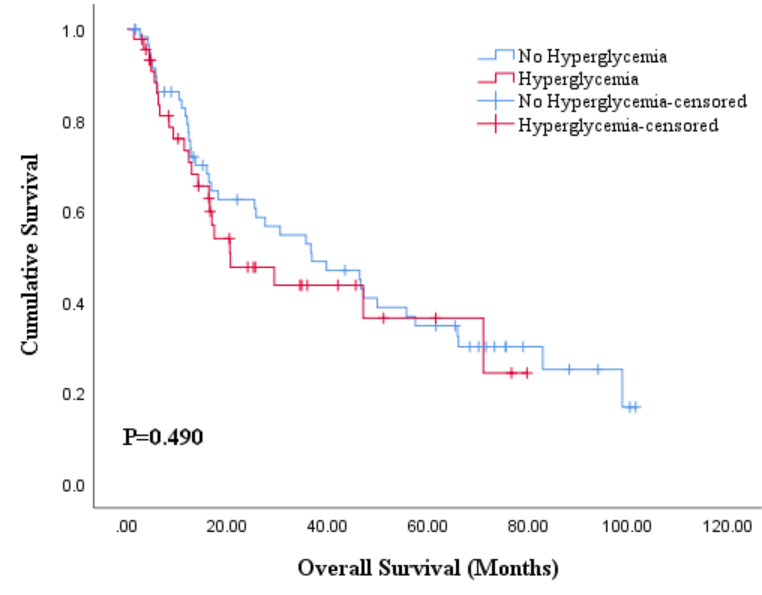**  D |
| **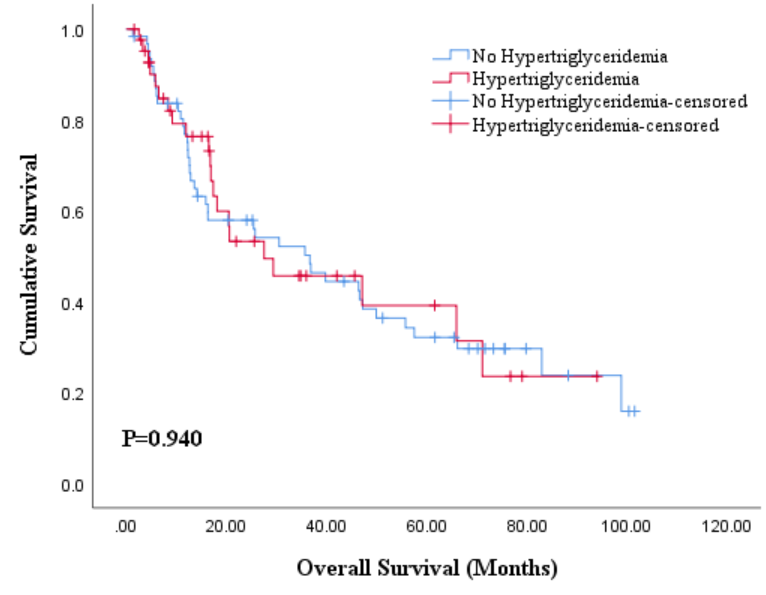**  F  E | **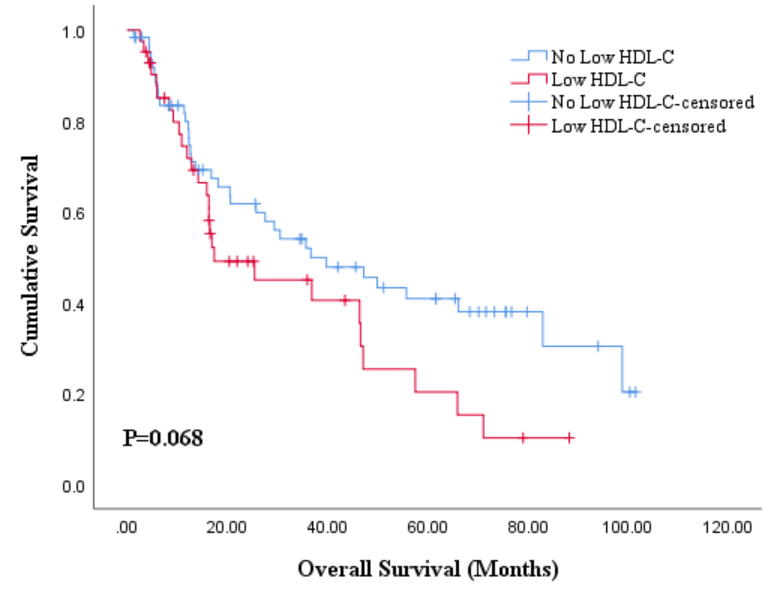** |

**Supplementary Figure 3** Kaplan-Meier survival analysis of the OS stratified by MetS and its components in MIBC patients after PSM. **(A)** MetS and non-MetS; **(B)** BMI <25 and BMI ≥25; **(C)** hypertension and no hypertension; **(D)** hyperglycemia and no hyperglycemia; **(E)** hypertriglyceridemia and no hypertriglyceridemia; **(F)** low HDL-C and no low HDL-C. BMI, body mass index; HDL-C, high density lipoprotein cholesterol; MetS, metabolic syndrome; MIBC, muscle invasive bladder cancer; OS, overall survival; PSM, propensity score match.

A

| **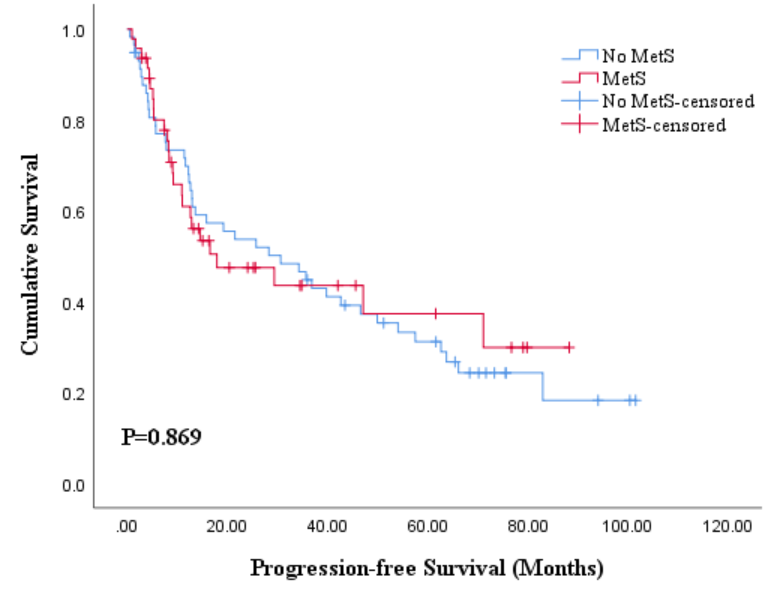**  B | **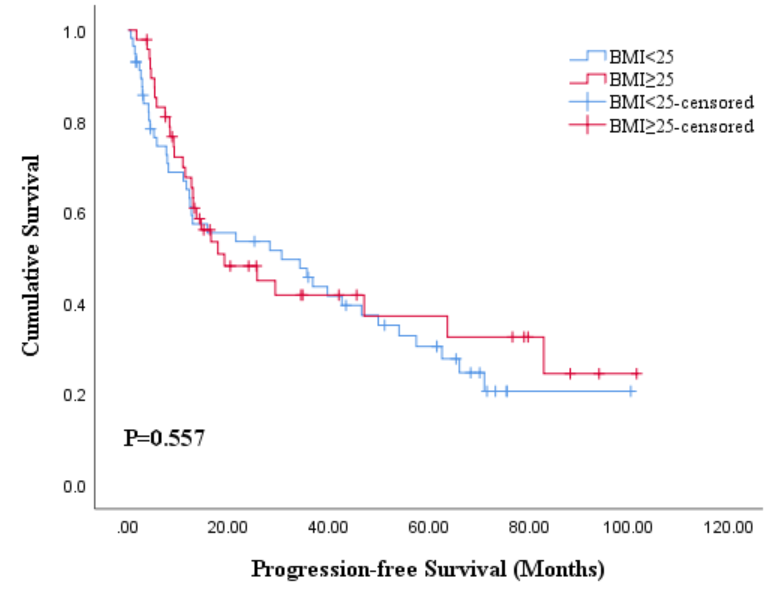** |
| --- | --- |
| **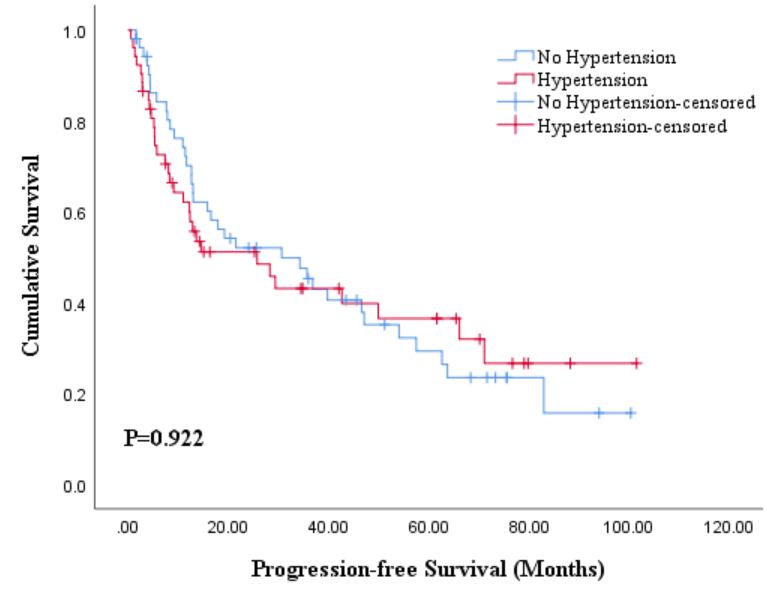**  C  D | **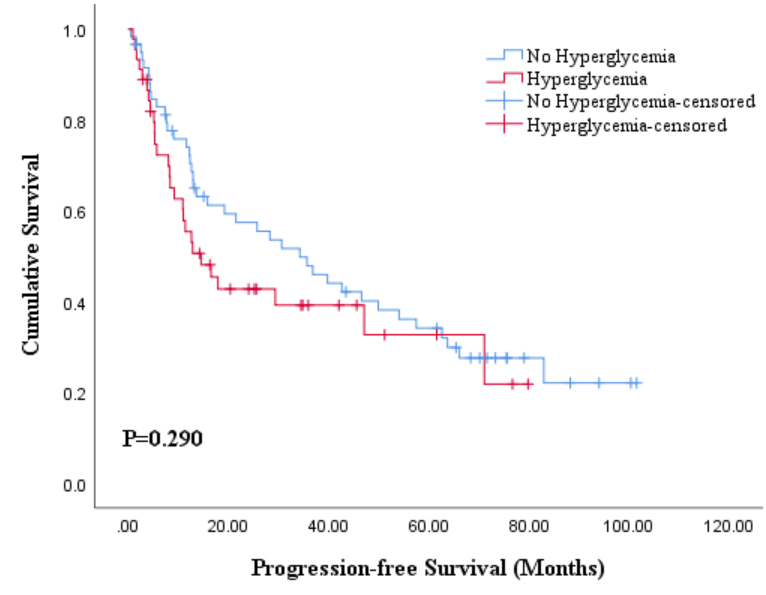** |
| **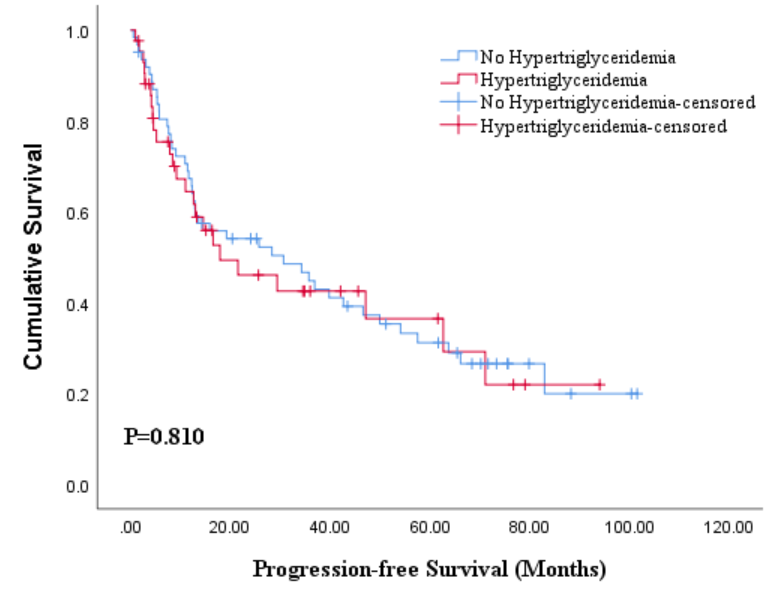**  E  F | **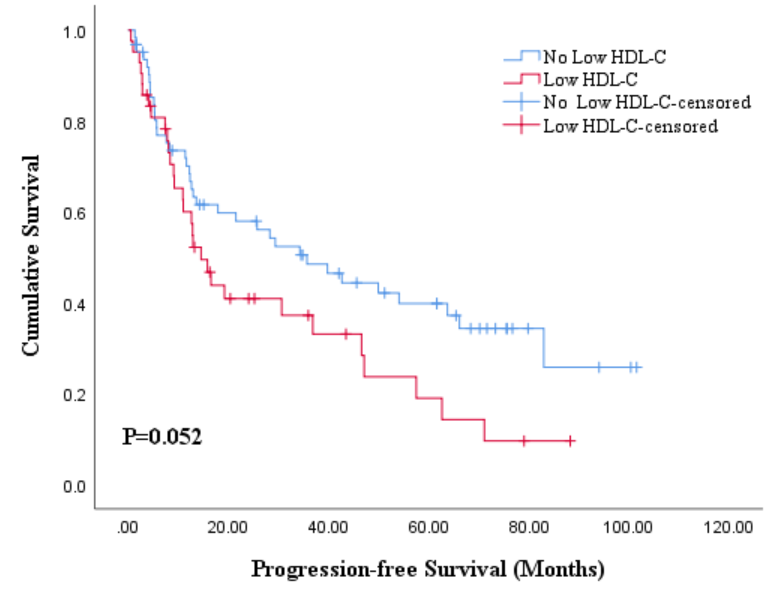** |

**Supplementary Figure 4** Kaplan-Meier survival analysis of the PFS stratified by MetS and its components in MIBC patients after PSM. **(A)** MetS and non-MetS; **(B)** BMI <25 and BMI ≥25; **(C)** hypertension and no hypertension; **(D)** hyperglycemia and no hyperglycemia; **(E)** hypertriglyceridemia and no hypertriglyceridemia; **(F)** low HDL-C and no low HDL-C. BMI, body mass index; HDL-C, high density lipoprotein cholesterol; MetS, metabolic syndrome; MIBC, muscle invasive bladder cancer; PFS, progression-free survival; PSM, propensity score match.
